# Gender Differences in Interpersonal Problems of Alcohol-Dependent Patients and Healthy Controls

**DOI:** 10.3390/ijerph6123010

**Published:** 2009-12-01

**Authors:** Sandra E. Mueller, Bigna Degen, Sylvie Petitjean, Gerhard A. Wiesbeck, Marc Walter

**Affiliations:** Division of Substance Use Disorders, Psychiatric Hospital of the University of Basel, Wilhelm Klein-Str. 27, CH-4025 Basel, Switzerland; E-Mails: Bigna.degen@upkbs.ch (B.D.); Sylvie.petitjean@upkbs.ch (S.P.); Gerhard.wiesbeck@upkbs.ch (G.W.); Marc.walter@upkbs.ch (M.W.)

**Keywords:** alcohol dependence, gender differences, interpersonal problems, personality, sex differences

## Abstract

Alcohol dependence is a heavy burden on patients, their families, and society. Epidemiological studies indicate that alcohol dependence will affect many individuals at some time in their lives, with men affected more frequently than women. Since alcohol-dependent patients often exhibit a lack of social skills and suffer from interpersonal problems, the aim of this study is to elucidate whether men and women experience the same interpersonal problems. Eighty-five alcohol-dependent patients (48 men; 37 women) after detoxification and 62 healthy controls (35 men; 27 women) were recruited. Interpersonal problems were measured with the Inventory of Interpersonal Problems (IIP-64). Additionally, alcohol-dependent patients were interviewed with the Alcohol Use Disorders Identification Test (AUDIT) and were subtyped according to Lesch’s Alcohol Typology (LAT). There were no significant gender differences in the AUDIT and LAT between alcohol-dependent men and women. Interpersonal problems of alcohol-dependent men differed significantly in one out of eight dimensions from controls; alcohol-dependent men perceive themselves as colder than male controls. Alcohol-dependent women differed in four out of eight interpersonal dimensions from female controls. Alcohol-dependent women rated themselves as significantly more vindictive, more introverted, more overly accommodating and more intrusive than female controls. Results suggest that alcohol-dependent men and women suffer from different interpersonal problems and furthermore alcohol-dependent women perceive more interpersonal problems, whereas the severity of alcohol dependence did not differ between the groups. Our findings indicate that alcohol-dependent women may profit more from a gender-specific treatment approach aimed at improving treatment outcome than alcohol-dependent men.

## 1. Introduction

Research on alcoholism initially focused predominantly on alcohol-dependent men, resulting in under-representation of women with alcohol use disorder. The first review on gender differences in alcoholism was 1992 by Jarvis and colleagues [1]; since then gender specific research in alcoholism has gained a lot of attention.

Overall men are more likely to suffer from alcohol use disorders [2]; however, relapse rates and time to relapse are similar across the genders [3]. A few studies have reported that women and men often begin treatment with similarly severe alcohol problems [4–6] but that women are more likely to have poor prognostic characteristics for treatment outcome [6,7]. In a study by Ross and colleagues [8], alcohol-dependent men reported drinking greater absolute amounts of alcohol, with earlier onset of heavy drinking than women [9]. However, there were no gender differences with respect to the frequency of binge drinking or in indicators of tolerance. In other findings, women reported more abstinent days and fewer drinks per day than men 3 months prior to the baseline measurement However, it appeared that women were heavier drinkers than men with respect to the index of drinking to intoxication [10].

Social stressors or reasons for drinking seem to differ more than the actual characteristics of drinking across gender. For alcohol-dependent men, marriage appears to be a protective factor; when men with an alcohol disorder drink, this is likely to create marital stress. In contrast, alcohol-dependent women appear to be at greater risk if married or as a result of marital stress [3,11]. This may reflect the fact that women are more likely than men to have a spouse or partner who drinks more than they do [12]. Further, women are more likely to relapse with a romantic partner or female friends—in contrast to alcohol-dependent men, who are more likely to relapse when alone [3,10]. Overall, men with an alcohol problem had greater exposure to peers’ drinking and women had greater exposure to partner’s drinking [13].

A study investigating the reasons of problem drinkers compared to non-problem drinkers found that women were more likely to have experienced family and interpersonal problems, the death of someone close and emotional distress that may lead to drinking, while men were more likely to have experienced workplace problems [13]. A representative sample of drinkers in Finland revealed that women were more likely to report that drinking had helped them to sort out interpersonal problems at home or in the workplace, to feel more optimistic about life, and to express their feelings, in contrast to men who reported more commonly that drinking helped them to be funnier and wittier and to get closer to the opposite sex. Overall, men tended to perceive more hedonic benefits while women perceived more functional benefits of drinking [14]. Factors preceding relapse episodes differ between the genders, in that women are more likely to drink in response to negative emotional states and interpersonal influences [15,16] while men, on the contrary, are more likely to relapse as a result of positive affect [15] and social pressure [16]. Other findings suggest that alcohol-dependent subjects of both genders experience predominantly negative moods. Immediately after the relapse, both genders reported a mix of negative and positive moods, with a tendency for men to report more positive moods after drinking than women [10]. These gender differences of positive and negative mood and intra- versus interpersonal conditions are expected to be reflected in Marlatt’s taxonomy of high risk situations for relapse; however no gender difference was found [10].

A recent study found that the association that women report drinking more than men in response to unpleasant emotions and conflicts with others seems to be mediated by severity of depression [17]. Women with drinking problems report more depression, more psychiatric problems and are more likely to drink to relieve negative affect [18], which is supported by an European epidemiological study showing that alcohol-dependent women have a higher overall rate of co-morbid psychiatric disorders than men, especially affective disorders [19]. Two studies on gender differences in relapse to alcohol found that, at baseline, women scored higher on depressive symptomatology than men [6,7].

Alcohol-dependent patients differ from controls in coping styles and personality characteristics, while alcohol-dependent females differ greatly in terms of coping styles, personality variables and in terms of conflicts [20]. Especially for alcohol-dependent women, interpersonal conflicts appear to be an additional risk factor [3]. Problems in interpersonal relationships lead to frustration in interactions, to psychological distress and to lower quality of life, which in turn enhance the aforementioned negative social consequences and lead to maintained substance use [21], which then sustains interpersonal problems. To our knowledge, very few studies have addressed gender differences in interpersonal problems. One study investigating healthy controls failed to reveal any difference in the subscales of the Inventory of Interpersonal Problems by gender [22].

A large body of literature in alcoholism has concentrated on personality traits and it has been found, for example, that novelty seeking is a strong predictor for relapse [23–25]. Nevertheless, no integrative pattern for dependence (such as an addictive personality) could be found, and this in turn has led to the development of various alcohol typologies, such as Barbor’s [26] or Cloninger’s [27] typology of alcoholism. Even if the objective of all alcohol typologies is not to categorize, but to provide help in assessing the course and prognosis [28], opinions on the validity of these typologies differ considerably. Most authors emphasize that dichotomous typologies are unlikely to be complex enough to prove helpful in clinical work [29]. Therefore, more detailed methods are required to describe the interpersonal behavior style of these subjects. Dimensional approaches rather than dichotomous typologies are useful for clinical work. The strengths of the IIP-64 are that it uses a dimensional approach and assesses multiple aspects of interpersonal functioning. Patients are not allocated to single categories, but to a specific region of the underlying circumplex model, e.g., the friendly-submissive region of the circumplex, which describes the patient’s distress in interpersonal interactions more precisely.

Therefore, the aim of the present study was to investigate self-perceived interpersonal problems in alcohol-dependent subjects, compared to healthy controls. The study was designed to test the following hypotheses: (1) Alcohol-dependent patients exhibit more self-perceived interpersonal problems than healthy controls, and (2) alcohol-dependent females suffer from different interpersonal problems than alcohol-dependent males. This is expected to be due to gender differences in alcoholism with respect to triggers to relapse, comorbid psychopathology, and socioeconomic variables, as mentioned above.

## 2. Methods

### 2.1. Participants and Procedures

The experimental sample consisted of 85 alcohol-dependent inpatients (48 males; 37 females) recruited at an alcohol detoxification unit after alcohol withdrawal. All patients had been diagnosed as alcohol dependent according to the DSM-IV criteria [30], without any other substance use disorder except tobacco dependence. The diagnosis of a current depressive episode was distributed equally between men (n = 15, 31.3%) and women (n = 14, 37.8%). After the completed alcohol detoxification at the Alcohol Treatment Unit of the Psychiatric Hospital of the University of Basel, patients were asked to participate in a questionnaire study. Patients were interviewed by a psychologist or an assistant doctor, using three questionnaires. Questionnaires were filled out in the following sequence: First, Lesch’s Alcohol Typology (LAT), the Alcohol Use Disorder Identification Test (AUDIT), and afterwards the Inventory of Interpersonal Problems (IIP-64) in paper-and-pencil format. The procedure lasted about 50 minutes. All patients provided written informed consent. The control group consisted of 62 healthy participants (35 males; 27 females) working in the health sector and were eligible if their age was between 18 and 65 years. They only filled out the Inventory of Interpersonal Problems (IIP-64) and provided written informed consent.

### 2.2. Materials and Measures

The Lesch Alcohol Typology (LAT) categorizes alcohol-dependent subjects into 4 subtypes, on the basis of various questions, such as family history characteristics, personal psychopathology and substance use history. Type I alcohol dependents exhibit very intense alcohol withdrawal syndrome and very intense alcohol dependence, with less other psychopathology, the so-called model of “allergy”. Type II alcohol-dependent patients use alcohol as a self-medication because of its anxiolytic effects and try to reduce anxiety or conflicts. The main characteristics of the Type III alcohol dependents are depressive symptoms to which the alcohol is used as a self-medication. And last but not least, Type IV alcohol dependents show pre-morbid cerebral defects or behavioral disorders predominantly in childhood, the so-called “alcohol drinking as adaptation” model [31,32].

The AUDIT is composed of 10 questions examining the quantity and frequency of alcohol drinking and alcohol-related behaviors and consequences, in which a score of 8 or more indicates that problematic alcohol use is suspected. A high AUDIT score is related to greater severity of alcohol dependence [33].

The German version of the Inventory of Interpersonal Problems (IIP-64) is a 64-item questionnaire used to assess self-perceived distress in interpersonal relationships [34]. The scales are arranged in a circumplex model, where two orthogonal dimensions, affiliation and dominance, are the main axes. Adjacent scales have more similarity and opposite scales have opposite qualities. The horizontal axis describes how much friendliness a person displays toward someone else and refers to nurturance, love, or affiliation, where the anchor on the right end is *excessively nurturant* and that on the left end is *cold*, referring to hostility, coldness and hate. The vertical axis quantifies the power or control someone else claims, and refers to status, agency or dominance, where the anchor on the upper end is *domineering* and the anchor on the lower end is *submissive*. Counterclockwise from the top of the circle, these subscales include: (1) *domineering, i.e.*, being too controlling or manipulative in interpersonal interactions; (2) *vindictive, i.e.*, being frequently egocentric and hostile in dealing with others; (3) *cold*, *i.e.*, having minimal feelings of affection for, and little connection with other people; (4) *socially avoidant*, *i.e.*, being socially avoidant and anxious and having difficulties approaching others; (5) *submissive*, *i.e.*, having difficulties expressing one’s needs to others; (6) *exploitable*, *i.e.*, being gullible and easily taken advantage of by people; (7) *overly nurturant*, *i.e.*, being excessively selfless, generous, trusting and caring; and (8) *intrusive*, *i.e.*, imposing one’s needs and having difficulties respecting the personal boundaries of other people. Due to the two main axes of love and dominance, the circumplex model can be divided into four regions; going clockwise, these are a friendly-dominant, a friendly-submissive, a hostile-submissive and a hostile-dominant region.

### 2.3. Statistical Analysis

Data analyses included the χ^2^ test for categorical variables and non-parametric tests for ordinal data. If normal distribution was given for continuous variables, appropriate analyses such as one-way ANOVA were used.

The raw data of the IIP-64 were first transformed to z-scores to ensure normal distribution. As after the z-transformation, some of the IIP-64 dimensions still failed to exhibit normal distribution, the p-level was adjusted to p = 0.010 for a more conservative analysis. Because of the intercorrelation of the IIP-64 dimensions, a multivariate analysis MANOVA was chosen for the IIP-64 data. All statistical analyses were calculated using the statistical software SPSS version 14.0 for Windows.

## 3. Results

### 3.1. Demographic and Substance Use Characteristics

Age and gender were equally distributed between the groups. The alcohol-dependent sample had a mean age of 46 years (SD 9.5), compared to 43 years (SD 10.1) for the control sample. Gender distribution was equal in the two groups, with 48 males (56.5%) and 37 females (43.5%) in the alcohol-dependent group compared to 35 males (56.5%) and 27 females (43.5%) in the control group. There was also no significant difference between the groups with respect to age. Family status differed significantly among alcohol-dependent patients. While 20 alcohol-dependent males (24.1%) reported that they were unmarried, this was true for only five alcohol-dependent women (7.8%). No significant differences across gender were found for positive family history of alcoholism, tobacco dependence or psychiatric diseases (Table 1).

No differences across gender were found in self-reported variables, such as years of pathological drinking, the first experienced withdrawal symptom (in years), loss of control in the last 3 months or the longest sober period. Alcohol-dependent men did not differ significantly from alcohol-dependent women in the AUDIT. Furthermore, when every single question of the AUDIT was compared across gender with non-parametric tests, one out of ten questions differed significantly across gender. Question 4 “How often during the last year have you found that you were not able to stop drinking once you had started” was more frequently affirmed by alcohol-dependent women (Z = −1.97, p = 0.049). The analysis of the Lesch Typology revealed that almost 50% of all patients were classified as type 3 (49.2%), the anti-depressive model of drinking. This was followed by type 4 (23.5%), type 2 (22.4%), and type 1 (7.4%) No gender difference was found regarding Lesch’s typology (Table 1).

One or more accidents under the influence of alcohol were reported, with similar frequency across gender, while even violation of the law was significantly more often confirmed by alcohol-dependent men 20 (41.7%) than women 7 (19.4%) (χ^2^ = 4.66, p = 0.031). The question whether they ever experienced a depressive episode was significantly more often confirmed by alcohol-dependent women, with 32 (86.5%) versus 33 (68.8%) by men (χ^2^ = 3.65, one-tailed, p = 0.047). Sleeping disorders without the influence of alcohol was reported to be similar across genders. Moreover, there was no gender-dependent difference in the incidence of suicide attempts (one or more).

### 3.2. Interpersonal Problems

A MANOVA was performed on continuous variables of the IIP dimensions for the complete alcohol-dependent sample compared to the healthy control group; in a second step, MANOVA analyzes were performed for each gender separately. For the complete sample, an overall effect for the group was found (multivariate F(8,138) = 3.144, p = 0.003). Five out of eight univariate effects reached significance. All results are given in Table 2.

**Figure 1.**
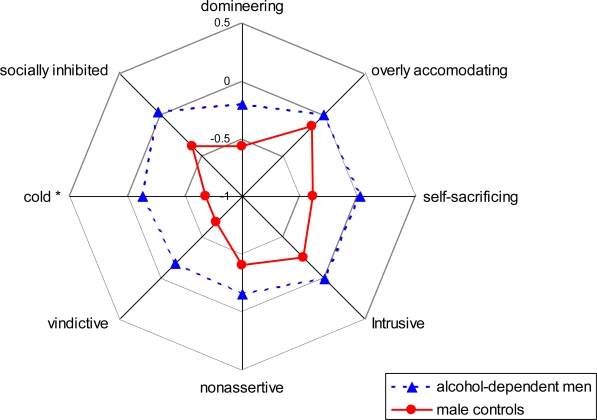
Circumplex model of interpersonal problems (IIP) of alcohol-dependent men *vs.* male controls (z-scores).

Firstly, a MANOVA of alcohol-dependent men versus healthy men was performed (Figure 1). The overall effect did not reach significance in the male group. There was one significant univariate effect for alcohol-dependent men, namely being too *cold* (F(1,82) = 7.404, p = 0.008).

**Figure 2.**
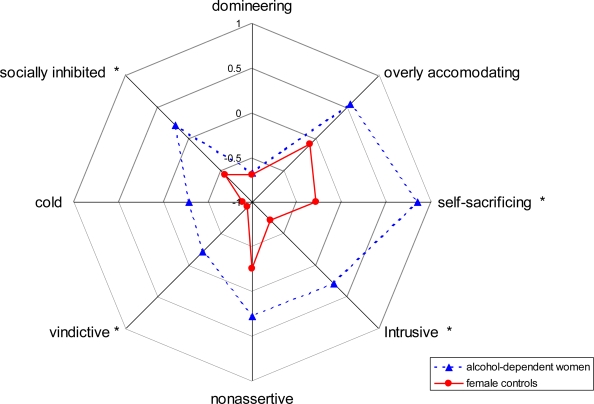
Circumplex model of interpersonal problems (IIP) of alcohol-dependent women *vs.* female controls (z-scores).

Secondly, a MANOVA of the alcohol-dependent women compared to female controls was performed (Figure 2). There was a significant overall effect in the female group (multivariate F(8,55) = 2.979, p = 0.008). Additionally, four out of eight dimensions in the univariate effects reached significance. Alcohol-dependent women had higher scores on being too vindictive (F(1,63) = 8.739, p = 0.004), being too socially inhibited (F(1,63) = 8.532, p = 0.005), too self-sacrificing (F(1,63) = 14.198, p < 0.001), and too intrusive (F(1,63) = 10.283, p = 0.002).

In a third step, the dichotomous variable of having a reported life-time depressive episode was used as a covariate in the MANOVA. No effect of this covariate could be found in either gender groups. Finally, the healthy control group alone was analyzed by a MANOVA for gender differences. No overall group effect for gender was found and none of the univariate dimensions reached significance.

## 4. Discussion

The current study revealed as an overall group effect that alcohol-dependent patients reported a higher severity of interpersonal problems than healthy controls. Further analyses revealed that this difference is mainly mediated by alcohol-dependent women, irrespective of the severity of their alcohol dependence. Alcohol-dependent women reported a higher burden in the dimensions of being too vindictive, too socially avoidant, too self-sacrificing and too intrusive compared to the female controls. Overall, it can be stated that alcohol-dependent women are in the friendly-submissive region, whereas alcohol-dependent men cannot be classified to any region of the IIP dimensions. In contrast, alcohol-dependent men did not differ in the overall effect from male controls; however, in the dimension of being too *cold* alcohol-dependent men scored significantly higher than male controls. Because no differences between male and female alcohol-dependent patients regarding a current depressive episode were found, the gender differences in interpersonal problems could not be mediated by current depressive episodes.

In this study, no differences across gender regarding characteristics of alcohol use could be found, except that women more frequently reported they failed to stop drinking once started. This finding is supported by the results of Rubin and colleagues [10], who reported that alcohol-dependent women drank more often to intoxication than their male counterparts, when gender and weight were taken into account. Another significant difference across gender was the family status. Alcohol-dependent men were more frequently single or unmarried compared to alcohol-dependent women. In this sample, alcohol-dependent women were more frequently in relationships, and prior research affirmed that women are more likely than men to have a spouse or partner who drinks even more than they do [12]. With this background, it may be assumed that alcohol-dependent women perceived more marital or family stress and more conflicts in the family, and that these aggravated interpersonal problems. This would in turn explain the present results of a higher burden of interpersonal problems in alcohol-dependent women.

Even if in this sample, the current depressive episodes did not differ across gender, although alcohol-dependent women reported more frequently life-time depressive episodes than alcohol-dependent men. This is in line with several findings that alcohol-dependent women score higher on depressive symptomatology at the beginning of treatment for alcohol and that comorbid psychiatric disorders—especially affective disorders—are more frequent in women with an alcohol use disorder [6,7,19]. Interestingly, the most frequent type of Lesch’s typology was Type III, the model of drinking for its anti-depressive effect, which exhibited no gender differences. This is contradicting to the finding of Sperling and colleagues [35] that alcohol-dependent women were more likely to be classified as Type III while alcohol-dependent men were more likely to be classified as Type IV of Lesch’s typology. In general, it is important to note that depressive episodes have negative influences on self-perception and interpersonal behavior. Nevertheless, findings for depressive patients with the IIP are somewhat inconsistent. Alden and Philips [36] found that depressive patients are comparable to controls, but Stangier *et al*. [37] reported that patients with a major depressive episode showed higher values on the subscales *socially avoidant*, *non-assertive*, *exploitable (overly accommodating)*, and *overly nurturant (self-sacrificing),* compared to the normative sample. The variable of life-time depressive episode was used as a covariate in the multivariate analysis and no effect was found. Furthermore there was no difference in current depressive episodes, so that it can be stated that the present results of the IIP dimensions are probably not affected by life-time or current depressive episodes.

It might be that the gender difference in perceived interpersonal problems is due to a lower self-image in alcohol-dependent women compared to alcohol-dependent men, as was found in a study of Aubry and colleagues [38]. A lower self-image and an overall higher depressive group of symptoms can lead to a biased perception, that the subject is the source of most mistakes.

One limitation of our study is that alcohol-dependent patients were recruited shortly after alcohol detoxification. Moreover, the findings should be corroborated with a larger sample size.

In summary, the most important finding of the present study is that especially alcohol-dependent women suffer from more interpersonal problems than men. This may be caused by the variety of preceding gender differences found in alcoholism research, which may lead to more perceived or effective interpersonal problems. Neither prior research [22] nor the present analysis of the control group found gender differences in interpersonal problems of healthy controls, suggesting that the difference is not due to gender per se, but may be linked to alcohol dependence or to the consequences of chronic alcoholism. Furthermore, a large body of literature shows that alcohol-dependent women, in contrast to alcohol-dependent men, suffer from or drink due to interpersonal problems [3,13–16], supporting the present finding. These limitations notwithstanding, the results of the current study retain some significant clinical implications. This is the first examination of gender differences in interpersonal problems of alcohol-dependent patients. It establishes that alcohol-dependent women suffer from different and more interpersonal problems than alcohol-dependent men, irrespective of the severity of alcohol dependence or affective disorder. This difference should be addressed in gender specific treatment programs to improve treatment outcome.

## Figures and Tables

**Table 1.** Characteristics of gender differences in the alcohol-dependent group.

		Alcohol-dependent men	Alcohol-dependent women	p
Gender distribution; n (%)		48 (56.5%)	37 (43.5%)	n.s.

Age		44.6 (10.6)	48.5 (7.4)	n.s.

AUDIT (total score)		24.2	26.8	n.s.
Family status				
Unmarried		20 (41.7%)	5 (13.9%)	
Living together		14 (29.2%)	16 (44.4%)	0.022
Separated/divorced		14 (29.2%)	15 (41.7%)	

Positive family history of				
Alcohol dependence	Yes	18 (37.5%)	17 (47.2%)	n.s.
	No	30 (62.5%)	19 (52.8%)	
Tobacco dependence	Yes	38 (79.2%)	29 (78.4%)	n.s.
	No	10 (20.8%)	8 (21.6%)	
Psychiatric diseases	Yes	13 (27.1%)	14 (38.9%)	n.s.
	No	35 (72.9%)	22 (61.1%)	

Lesch’s typology				
Type 1 (7.4%)		3 (6.3%)	1 (2.7%)	
Type 2 (22.4%)		9 (18.8%)	10 (27%)	n.s.
Type 3 (49.2%)		24 (50%)	18 (48.6%)	
Type 4 (23.5%)		12 (25%)	8 (21.6%)	

**Table 2.** Differences in interpersonal problems for the complete sample and separately for each gender.

	Alcohol-group (n = 85)	Healthy controls (n = 62)	Univ F	p
Domineering	−0.411	−0.628	2.51	0.115
Vindictive	−0.196	−0.788	14.32	>0.001
Cold	−0.203	−0.776	13.49	>0.001
Socially inhibited	0.111	−0.467	12.27	0.001
Non-assertive	0.034	−0.339	4.28	0.040
Overly accommodating	0.231	−0.228	3.68	0.057
Self-sacrificing	0.379	−0.343	14.49	>0.001
Intrusive	0.127	−0.456	9.71	0.002
Multivariate *F*(8,138) = 3.14, p = 0.003				
	Alcohol–dependent men (n = 48)	Male controls (n = 35)	Univ F	p

Domineering	−0.208	−0.573	4.25	0.042
Vindictive	−0.18	−0.685	5.85	0.018
Cold	−0.137	−0.686	7.40	0.008
Socially inhibited	0.032	−0.389	4.08	0.047
Non–assertive	−0.155	−0.405	1.34	0.251
Overly accommodating	−0.008	−0.144	0.386	0.536
Self–sacrificing	0.017	−0.390	3.18	0.078
Intrusive	0.005	−0.257	1.35	0.248
Multivariate F(8,74) = 1.07, p = 0.394					
	Alcohol–dependent women (n = 37)	Female controls (n = 27)	Univ F	p

Domineering	−0.674	−0.699	0.014	0.907
Vindictive	−0.216	−0.923	8.74	0.004
Cold	−0.29	−0.893	6.02	0.017
Socially inhibited	0.213	−0.569	8.53	0.005
Non–assertive	0.278	−0.253	3.12	0.082
Overly accommodating	0.54	−0.084	4.36	0.041
Self–sacrificing	0.849	−0.282	14.2	>0.001
Intrusive	0.286	−0.7133	10.28	0.002
Multivariate F(8,55) = 2.98, p = 0.008				
